# Prevalence and predictors of staff burnout at a tertiary cancer center in Jordan

**DOI:** 10.3934/publichealth.2025026

**Published:** 2025-04-15

**Authors:** Omar Shamieh, Waleed Alrjoub, Ghadeer Alarjeh, Khawlah Ammar, Mohammad Abu Hazim, Tayseer Shawash, Osama Zamel, Maysa Al-Hussaini, Majeda Al-Ruzzieh, Hikmat Abdel-Razeq, Asem Mansour

**Affiliations:** 1 Department of Palliative Care, King Hussein Cancer Center (KHCC), Amman, Jordan; 2 Faculty of Medicine, University of Jordan, Amman, Jordan; 3 Centre for Palliative and Cancer Care in Conflict (CPCCC), KHCC, Amman, Jordan; 4 Survey research unit, Office of Scientific Affairs and Research, KHCC, Amman, Jordan; 5 Psychosocial Oncology Program, KHCC, Amman, Jordan; 6 The Jordanian Centre for Psychological & Educational Consultation & Therapy, Amman, Jordan; 7 Department of Internal Medicine, KHCC, Amman, Jordan; 8 Department of Cell Therapy and Applied Genomics (CTAG), KHCC, Amman, Jordan; 9 Office of Nursing, KHCC, Amman, Jordan; 10 Department of Radiology, KHCC, Amman, Jordan

**Keywords:** burnout in oncology, emotional exhaustion, depersonalization, personal accomplishment, mental health, Jordan healthcare, Maslach Burnout Inventory (MBI), occupational stress, Patient Health Questionnaire (PHQ-9), job satisfaction

## Abstract

Burnout among oncology healthcare providers (HCPs) poses significant challenges to both personal well-being and patient care quality. To inform targeted interventions, this study assessed burnout prevalence and its predictors among HCPs in a tertiary cancer center in Jordan. A cross-sectional study was conducted from October 10, 2023, to April 14, 2024, using an online questionnaire available in both English and Arabic. The survey, distributed via email and social media, included the Maslach Burnout Inventory (MBI), Patient Health Questionnaire-9 (PHQ-9), and sociodemographic items. Descriptive statistics, binary logistic, and group comparisons analyzed the relationships between demographic/work characteristics, depression, and burnout. Of 996 respondents, 692 (69.4%) completed the survey. Most participants were male (54.6%) and Muslim (98.3%), with 41.6% aged under 30. Respondents included physicians (17.1%), nurses (28.0%), and other healthcare roles (54.9%). Burnout levels were high, with 75.7% reporting high emotional exhaustion (EE), 35.3% experiencing high depersonalization (DP), and 27.2% showing low personal accomplishment (PA). Binary logistic regression analysis identified significant predictors of high EE, including lower income (<500 JD ≈ 705 USD, *p* = 0.004), thoughts of quitting (*p* = 0.000), and lack of burnout training (*p* = 0.007). High DP was associated with a lack of hobbies (*p* = 0.003) and thoughts of quitting (*p* = 0.000), while low PA was linked to a higher patient caseload (*p* = 0.000). Elevated PHQ-9 scores, indicative of depression, were significantly correlated with increased burnout across all dimensions (*p* < 0.001). In conclusion, burnout is highly prevalent among oncology HCPs in Jordan, with strong associations between burnout and specific demographic and work-related factors. Targeted interventions emphasizing stress management, organizational support, and effective coping mechanisms are crucial to mitigate burnout and enhance job satisfaction among oncology staff.

## 1. Introduction

Healthcare professionals (HCPs), particularly those in oncology, face substantial challenges in managing the emotional and physical demands of caring for patients who experience pain and other distressing symptoms. Sustaining motivation can be difficult under these conditions, as oncology HCPs are at a heightened risk for compassion fatigue (CF) and burnout (BO), both of which significantly affect their well-being and performance. Oncology nurses, for instance, frequently experience CF due to prolonged exposure to patient trauma and the intense demands of cancer care [1]. Additionally, a growing body of evidence reveals a high prevalence of BO among HCPs, including oncologists, driven by chronic workplace stress [2]. Burnout, first conceptualized in the 1970s, is marked by emotional exhaustion, depersonalization, and a reduced sense of personal accomplishment [3].

CF and BO are often intertwined, as CF involves secondary traumatic stress resulting from empathetic engagement with patients' suffering, thereby heightening the risk of BO [4]. Maslach developed the Maslach Burnout Inventory (MBI) to assess and quantify BO, a widely validated tool encompassing three subscales: emotional exhaustion (EE), depersonalization (DP), and personal accomplishment (PA) [5]. BO not only affects the personal well-being of HCPs but also has substantial organizational implications, including decreased productivity, increased turnover, and elevated operational costs [6]–[8]. This cycle of inefficiency can ultimately compromise care quality and patient safety within healthcare settings [9].

The prevalence of BO among HCPs is high, with systematic reviews indicating rates as high as 80.5% globally [10]. Regional studies in the Arab world also show varying BO prevalence rates among HCPs, underscoring the need for tailored interventions within local contexts [11].

Sipos [12],[13] extensively investigated burnout among HCPs. His research highlighted the significant influence of organizational factors, including inadequate staffing (emphasized by high staff-to-patient ratios), poor leadership, and negative organizational culture, on burnout risk. Conversely, transformational leadership, open communication, teamwork, and recognition within a supportive environment can effectively mitigate burnout. Furthermore, his study during the COVID-19 pandemic identified additional contributing factors among oncology professionals, such as long working hours, on-call duties, and considering leaving the profession, which significantly impacted burnout levels, particularly regarding depersonalization and emotional exhaustion.

Other regional studies, such as those conducted among oncologists in the Middle East and North Africa (MENA) region [3],[12], have also identified age, workload, and levels of organizational support as significant factors influencing burnout prevalence, underscoring the multifactorial nature of burnout and the need for a comprehensive approach to address this critical issue among healthcare professionals.

Research on BO extends beyond oncologists to other HCPs, including oncology nurses, who also contend with high stress levels. A study conducted in Jordan found elevated levels of BO and CF among specialized oncology nurses, which were attributed to heavy workloads and low compassion satisfaction [4]. This highlights the critical role of organizational support in reducing BO and enhancing job satisfaction among HCPs. Similarly, research from Istanbul demonstrated significant correlations between BO, coping strategies, and job-related stress among oncology HCPs, with adaptive coping mechanisms proving protective against BO [14]. This suggests the value of promoting effective stress management strategies within healthcare settings.

The sociocultural context within Jordan and the broader Arab world, characterized by shared cultural values and prevalent religious beliefs, introduces an additional layer of burnout among HCPs. While faith and religious beliefs often provide resilience and coping resources to many people, the sociocultural pressure may foster an expectation of self-sacrifice, and many HCPs choose to persist in their roles despite experiencing burnout. This can discourage them from seeking help or openly expressing concerns, thereby hindering effective burnout management. For example, a qualitative study in Riyadh, Saudi Arabia, involving 14 medical residents, identified an extensive stigma surrounding burnout. Key themes included the “burnout stigma cycle” and a strong reliance on self-coping strategies, with participants often avoiding formal support mechanisms [15]. Abusanad [3] and Elbarazi [11] have documented that such cultural dynamics play a significant role in shaping BO experiences in Arab healthcare settings, adding complexity to BO management strategies. Despite the growing research on BO among HCPs globally, there remains a scarcity of studies specific to the Arab region, including Jordan. This gap limits the development of tailored interventions and support systems that consider the unique cultural and organizational contexts of Arab healthcare settings. Understanding the prevalence, contributing factors, and demographic correlates of BO among oncology HCPs in Jordan is essential to inform evidence-based strategies aimed at mitigating its impact.

This study investigates burnout among oncology HCPs at King Hussein Cancer Center (KHCC) in Jordan. By examining the prevalence, work-related factors, and demographic influences of BO, this research aims to contribute to the knowledge base on BO in oncology settings, provide insights for policy and practice improvements, and ultimately enhance the well-being of HCPs and patient care quality in Jordanian oncology centers.

## 2. Materials and methods

### 2.1. Study design

This cross-sectional study was conducted using an online questionnaire developed on SurveyMonkey®, available in both English and Arabic. The study aimed to assess burnout and mental health status among healthcare professionals (HCPs) at a specialized oncology center.

### 2.2. Study setting

The study was conducted at the King Hussein Cancer Center (KHCC) in Amman, Jordan. KHCC is a leading cancer treatment and research facility in the Middle East, operating under the King Hussein Cancer Foundation (KHCF) as a non-profit organization. It serves around 60% of Jordan's cancer cases and provides care for patients from various Arab countries. KHCC's accreditation by the Joint Commission International (JCI) as a disease-specific cancer center highlights its commitment to quality and patient safety standards [16].

### 2.3. Data collection

Data were collected via a survey distributed from October 10, 2023, to April 14, 2024, through official emails and social media platforms, including verified WhatsApp groups. The survey consisted of the Maslach Burnout Inventory (MBI) and the Patient Health Questionnaire (PHQ-9), along with a sociodemographic questionnaire. Informed consent was implied by participants reading the introductory information letter and completing the survey voluntarily. Responses were anonymized to ensure confidentiality.

### 2.4. Participants and procedure

The study participants included a wide range of HCPs from KHCC, such as nurses, physicians, respiratory therapists, physiotherapists, pharmacists, occupational therapists, psychologists, social workers, and other professionals directly involved in patient care.

Participants accessed the survey through a secure link. The survey was structured into sections gathering sociodemographic and work-related characteristics (age, gender, marital status, socioeconomic status, children, religion, hobbies, occupation, work setting, experience with oncology patients, weekly caseload, and considerations about leaving oncology). The validated MBI and PHQ-9 instruments were used to measure burnout and mental health, respectively. The survey's anonymity was maintained, and no compensation was offered for participation.

The study received an Institutional Review Board (IRB) approval from KHCC in Amman, Jordan (IRB No. 23KHCC74). Participants were not compensated for their involvement, and the study was not funded.

### 2.5. Questionnaires

### 2.5.1. The Patient Health Questionnaire-9 (PHQ-9) [17]

The PHQ-9 is a widely used, validated, reliable, and self-administered tool designed to detect psychological disorders, particularly major depressive disorder (MDD). Developed in 1999 as an enhancement of the original PRIME-MD, the PHQ-9 is known for its high sensitivity and specificity. The questionnaire consists of nine items that measure the frequency of depressive symptoms experienced over the past two weeks, with each item scored on a scale from 0 (not at all) to 3 (nearly every day). Total scores range from 0 to 27.

The PHQ-9 employs two distinct algorithms: one for diagnosing depressive disorders and another for assessing the severity of depressive symptoms. The diagnostic algorithm, based on the Diagnostic and Statistical Manual of Mental Disorders, Fourth Edition (DSM-IV), differentiates between major depressive disorder (MDD) and other forms of depression. The severity algorithm classifies respondents according to their symptom severity as follows: none (0), minimal (1–4), mild (5–9), moderate (10–14), moderately severe (15–19), and severe (20–27). Depression is classified as major depression or other depression using the PHQ-9 diagnostic algorithm, or by a PHQ-9 score of 10 or higher [17].

Additionally, the PHQ-9 includes a functional health assessment that inquires how emotional difficulties affect work, home life, or relationships. The tool has been translated and validated in Arabic [18], and it should be used in conjunction with clinical judgment for accurate diagnosis and treatment planning (Table 1).

**Table 1.** Total score interpretation of PHQ-9.

**Total score**	**Depression severity**
**1–4**	Minimal depression
**5–9**	Mild depression
**10–14**	Moderate depression
**15–19**	Moderately severe depression
**20–27**	Severe depression

Note: Sourced from [17].

### 2.5.2. Maslach Burnout Inventory (MBI) [19]

The Maslach Burnout Inventory-Human Services Survey (MBI-HSS) consists of 22 items that assess burnout across three dimensions: emotional exhaustion (EE) with 9 items, depersonalization (DP) with 5 items, and personal accomplishment (PA) with 8 items. Respondents rate each item on a six-point Likert scale ranging from 0 (never) to 6 (daily). Scores are categorized as follows: for EE, high (>30), moderate (18–29), and low (≤17); for DP, high (≥12), moderate (6–11), and low (≤5); and for PA, high (≤33), moderate (34–39), and low (≥40). Elevated EE and DP scores and reduced PA scores indicate high levels of burnout [19] (Table 2).

**Table 2.** Scoring key of the Maslach Burnout Inventory Questionnaire (MBI).

**Burnout level**	**Emotional exhaustion**	**Depersonalization**	**Personal accomplishment**
**High**	≥27	≥10	0–33
**Moderate**	19–26	6–9	34–39
**Low**	0–18	0–5	≥40

Note: Sourced from [19].

### 2.5.3. Additional items

Two free-text questions were included to explore the sources of stressors and coping mechanisms used by participants. The questions were as follows:

Q1: What are the sources of stressors you encounter in your current work?

Q2: How do you cope with these stressors?

### 2.6. Translation and cross-cultural adaptation

Initially, we utilized the validated Arabic version of the MBI published by Sabbah [20]. This version demonstrated high internal consistency among Lebanese nurses, with Cronbach's alpha values of 0.89 for EE, 0.82 for DP, and 0.80 for PA. Given the cultural and professional similarities between the Lebanese healthcare setting and our study at the King Hussein Cancer Center (KHCC), leveraging this existing version provided a strong foundation. However, to further enhance the cultural relevance within the Jordanian context, we conducted a formal translation process.

To ensure cultural appropriateness and linguistic accuracy, we followed the EORTC-QLG Translation Guidelines [21] for the translation and cross-cultural adaptation of the Maslach Burnout Inventory (MBI). The process included forward translation by two independent bilingual translators (a palliative care consultant and a psychologist) who translated the MBI from English to Arabic, ensuring grammatical accuracy and comprehensibility. A third bilingual expert (a medical oncologist) reviewed the translations to create a reconciled version, combining the best elements of both translations. Back-translation by two additional bilingual translators (a psychologist and a social worker) translated the reconciled version back into English. Finally, an expert committee, including healthcare professionals and senior researchers, reviewed all translations and discrepancies, reaching a consensus to finalize the provisional Arabic version of the MBI.

### 2.7. Pilot testing

The provisional Arabic version of the MBI was pilot tested on 20 staff members from the KHCC to evaluate its clarity, acceptability, and relevance. Participants completed the questionnaire and provided feedback through structured interviews on any difficulties or ambiguities in the questions. Their feedback indicated that no changes or refinements were necessary for the final version of the questionnaire. This outcome confirms that the questionnaire is well suited for the target population, ensuring it is clear, relevant, and easily understood by the participants.

Furthermore, we conducted a thorough review and comparison of our translated version with the previously validated Arabic version by Sabbah [20]. This comparative analysis revealed no significant discrepancies, further supporting the content validity of our translated version.

### 2.8. Statistical analysis

Data were analyzed using SPSS 28. The demographic characteristics of participants were summarized using frequencies and percentages. This included data on gender, age groups, marital status, income brackets, smoking status, occupation, and years of experience. Mean, standard deviation, and range were computed for continuous variables. The MBI and PHQ-9 scores were analyzed using scoring guidelines to categorize levels of burnout (EE, DP, and PA) and depression severity.

The relationship between demographic/work characteristics and mental health outcomes (depression scores from PHQ-9 and burnout scores from MBI) was evaluated by binary logistic regression analyses to explore the predictors of depression and burnout among participants. Independent variables included age, gender, years of experience, patient load per week, and any other relevant demographic or work characteristic. We used independent sample t-tests and one-way ANOVA to compare PHQ-9 and MBI scores across different participant categories (e.g., gender, marital status, occupation type). A *p*-value < 0.05 was considered statistically significant.

## 3. Results

A total of 996 participants were enrolled in the survey between October 10, 2023, and April 14, 2024, responding to both the Arabic and English versions. Of these, 7 participants did not complete the demographic section, 234 did not complete the MBI, and 63 did not complete the PHQ-9. Ultimately, 692 participants completed all survey items and were included in the final analysis.

### 3.1. Demographic characteristics

The study surveyed 692 participants, with males comprising a slight majority (54.6%, *n* = 378). The largest age group was under 30 years old (41.6%, *n* = 288), and the vast majority identified as Muslim (98.3%, *n* = 680). More than half of the participants reported engaging in hobbies (53.5%, *n* = 367), and the majority were married (58.4%, *n* = 403). A slight majority of participants reported having children (54.3%, *n* = 375), with the largest income bracket being 501–1000 Jordan Dinars (40.8%, *n* = 282). Smoking was reported by 15.8% of participants (*n* = 109). In terms of occupation, 17.1% were physicians (*n* = 118), 28.0% were nurses (*n* = 193), and 54.9% fell into other occupational categories. The majority worked in the inpatient setting (61.2%, *n* = 423), and 45.1% (*n* = 344) had worked for less than 5 years in oncology. Patient load per week varied, with 43.1% (*n* = 296) handling more than 50 patients. Regarding job retention, 24.9% of participants reported always thinking about quitting (*n* = 169). Only 19.7% of participants reported receiving training on burnout (*n* = 136), while the majority (80.3%, *n* = 555) had not (Table 3).

**Table 3.** Demographic characteristics (*n* = 692).

Baseline characteristics	*N* (%)
**Gender**	
Male	378 (54.6)
Female	314 (45.4)
**Age group**	
Less than 30 years	288 (41.6)
31–40	230 (33.2)
41–51	133 (19.2)
Above 50	41 (5.9)
**Religion**	
Muslim	680 (98.3)
Christian	7 (1.0)
Others	5 (0.7)
**Engage in a hobby**	
Yes	367 (53.5)
No	319 (46.5)
**Marital status**	
Single	254 (36.8)
Married	403 (58.4)
Divorced	30 (4.3)
Widowed	3 (0.4)
**Having children?**	
Yes	375 (54.3)
No	315 (45.7)
**Monthly income (Jordanian Dinar)**	
Less than 500	226 (32.7)
501–1000	282 (40.8)
1001–1500	82 (11.9)
More than 1500	101 (14.6)
**Smoking**	
Yes	109 (15.8)
No	581 (84.2)
**Occupation**	
Physician	118 (17.1)
Nurse	193 (28.0)
Others	379 (54.9)
**Working area**	
Inpatient	423 (50.7)
Outpatient	236 (28.3)
Radiology department	68 (8.1)
Radiotherapy department	35 (4.2)
Pharmacy	43 (5.1)
Laboratory	30 (3.6)
**Duration of working in oncology (years)**	
Less than 5 years	344 (45.1)
6–10 years	163 (23.7)
11–15 years	81 (11.8)
More than 15 years	99 (14.4)
**Patient case load/week**	
20 or less	191 (27.8)
21–30	87 (12.7)
31–40	65 (9.5)
41–50	47 (6.9)
More than 50	296 (43.1)
**Thinking about quitting your job?**	
Always	169 (24.9)
Sometimes	274 (40.3)
Seldom	91 (13.4)
Never	146 (21.5)
**Training received on burnout**	
Yes	136 (19.7)
No	555 (80.3)

Note: Values are presented as frequencies (*n*) and percentages (%) of the total sample size. Distribution of working areas during the study period, with percentages based on total working areas due to participant rotations.

### 3.2. Patient Health Questionnaire-9 (PHQ-9)

**Figure 1.**
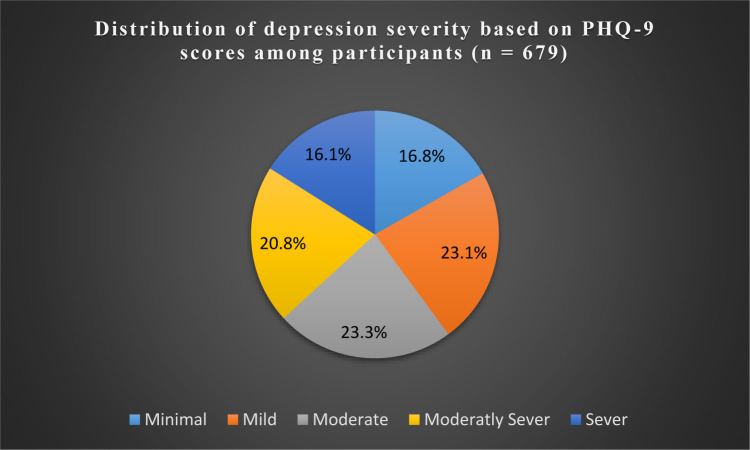
Level of depression based on the level of depressive symptom severity.

Figure 1 highlights a substantial portion of the sample experiencing varying degrees of depressive symptoms among the respondents. According to the PHQ-9 scores, moderate depressive symptoms were observed in 23.3% (*n* = 158) of respondents. Additionally, 20.8% (*n* = 141) had moderately severe symptoms, and 16.1% (*n* = 109) reported severe depressive symptoms. Overall, 54.5% (*n* = 370) of participants had a PHQ-9 score of 10 or higher, indicating the presence of clinically significant depressive symptoms. These results were categorized following the framework established by Kroenke and colleagues [17].

### 3.3. MBI components and burnout level

**Figure 2.**
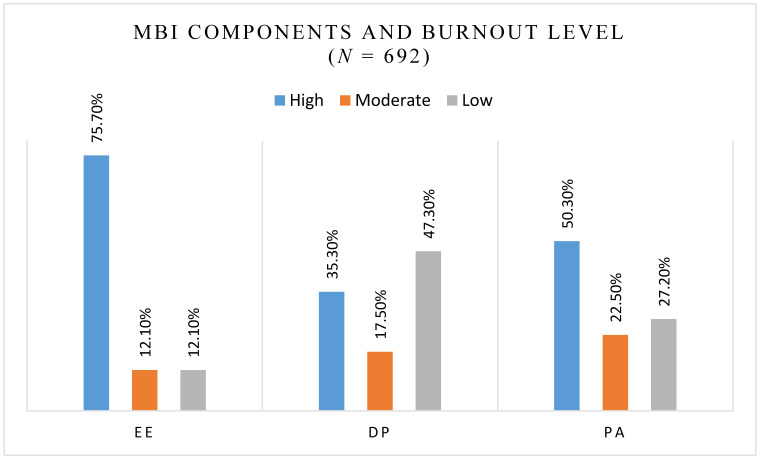
MBI components and burnout level.

Figure 2 displays the classification of MBI scores, illustrating the distribution of EE, DP, and PA across different burnout levels, The majority of participants (75.7%) experienced high levels of emotional exhaustion, indicating a significant prevalence of burnout in this dimension. Both moderate and low levels of EE were equally represented at 12.1%. A lower percentage of participants (35.3%) experienced high levels of depersonalization, while nearly half (47.3%) experienced low levels, suggesting that while emotional exhaustion is high, depersonalization is less prevalent.

About half of the participants (50.3%) reported high levels of personal accomplishment. However, 27.2% reported low levels, indicating that a considerable portion felt less accomplished in their work.

#### 3.3.1. Factors affecting the risk of developing EE, DP, and low PA

The results of a binary logistic regression analysis identified key factors associated with the risk of experiencing high levels of EE and DP and low levels of PA among oncology staff. Factors examined included demographic variables, lifestyle factors, professional experience, workload, and attitudes toward work. Significant associations (*p* < 0.05) between characteristics and burnout components are highlighted, with both the raw *p*-values and adjusted *p*-values (Adj *p*) displayed for each factor (Table 4).

#### 3.3.2. Emotional exhaustion (EE)

Gender appeared to influence EE, with female oncology staff exhibiting slightly higher EE levels (81.2%) compared to males (71.2%). However, this difference became non-significant when adjusting for other variables (Adj *p* = 0.283), suggesting that gender alone may not drive EE once broader factors are considered.

Interestingly, the age group showed a notable trend in which EE levels were highest among younger staff, especially those under 30 (85.1%), with levels gradually decreasing across older age groups (61% for those over 50). This trend is initially significant (*p* < 0.001), although it weakens upon adjustment (Adj *p* = 0.128), indicating that while younger age correlates with increased EE, other intertwined factors might modulate this relationship.

**Table 4.** Binary logistic regression analysis of predictive factors for developing emotional exhaustion (EE), depersonalization (DP), and personal accomplishment (PA).


Characteristics	EE			DP			PA		

	High *N* (%)	*Sig*	Adj *P*	High *N* (%)	*Sig*	Adj *P*	Low *N* (%)	*Sig*	Adj *P*

**Gender**		**0.002**	0.283		0.244	*NS*		0.864	*NS*
Male	269 (71.2)			126 (33.3)			104 (27.5)		
Female	255 (81.2)			118 (37.6)			84 (26.8)		
**Age group**		**0.000**	0.128		**0.000**	0.584		0.835	*NS*
Less than 30	245 (85.1)			134 (46.5)			82 (28.5)		
31–40	169 (73.5)			73 (31.7)			61 (26.5)		
41–50	85 (63.9)			30 (22.6)			36 (27.1)		
More than 50	25 (61)			7 (17.1)			9 (22)		
**Practicing hobby**		**0.008**	0.053		**0.004**	**0.003**		0.736	*NS*
Yes	262 (71.4)			111 (30.2)			102 (27.8)		
No	256 (80.3)			131 (41.1)			85 (26.6)		
**Marital status**		**0.000**	0.427		**0.000**	0.190		**0.005**	0.055
Single	214 (84.3)			117 (46.1)			85 (33.5)		
Married	287 (71.2)			118 (29.3)			90 (22.3)		
Divorced	21 (70)			7 (23.3)			10 (33.3)		
Widowed	1 (33.3)			1 (33.3)			2 (66.7)		
**Having children**		**0.000**			**0.000**			**0.024**
No	268 (85.1)			142 (45.1)			99 (31.4)		
Yes	254 (67.7)			102 (27.2)			89 (23.7)		
**Monthly income (JD)**		**0.000**	**0.004**		**0.000**	0.135		0.225	*NS*
≤500	171 (75.7)			65 (28.8)			64 (28.3)		
501–1000	226 (80.1)			124 (44)			66 (23.4)		
1001–1500	67 (81.7)			29 (35.4)			23 (28%)		
>1500	60 (59.4)			25 (24.8)			23 (33.7)		
**Smoking**		0.330	NS		0.331	NS		0.313	*NS*
Yes	87 (79.8)			43 (39.4)			34 (31.2)		
No	435 (74.9)			201 (34.6)			154 (26.5)		
**Years of experience**		0.083	NS		**0.003**	0.175		0.265	*NS*
<5 years	265 (77)			136 (39.5)			101 (29.4)		
6–10 years	128 (78.5)			59 (36.2)			36 (22.1)		
11–15 years	63 (77.8)			29 (35.8)			24 (29.6)		
>15 years	65 (65.7)			19 (19.2)			23 (23.2)		
**Number of pts/week**		0.179	NS		0.398	NS		**0.000**	**0.000**
Less than 20	144 (75.4)			68 (35.6)			74 (38.7)		
21–30	67 (77)			35 (40.2)			12 (13.8)		
31–40	43 (66.2)			27 (41.5)			18 (27.7)		
41–50	33 (70.2)			12 (25.5)			8 (17)		
More than 50	235 (79.4)			102 (34.5)			73 (24.7)		
**Think about quitting oncology work**		**0.000**	**0.000**		**0.000**	**0.000**		**0.013**	0.058
Always	158 (93.5)			101 (59.8)			61 (36.1)		
Sometimes	231 (84.3)			105 (38.3)			69 (25.2)		
Rarely	55 (60.4)			18 (19.8)			19 (20.9)		
Never	69 (47.3)			18 (12.3)			33 (22.6)		
**Had seminar/ workshop about burnout**		**0.001**	**0.007**		**0.009**	0.116		0.452	NS
Yes	87 (64)			35 (25.7)			33 (24.3)		
No	436 (78.6)			209 (37.7)			155 (27.9)		
**PHQ**		**0.000**			**0.000**			**0.000**	
Minimal	36 (31.6)			13 (11.4)			20 (17.5)		
Mild	103 (65.6)			31 (19.7)			40 (25.5)		
Moderate	130 (82.3)			60 (38)			33 (20.9)		
Moderately severe	137 (97.2)			63 (44.7)			42 (29.8)		
Severe	108 (99.1)			74 (67.9)			48 (44)		

Note: Statistical significance (*p* < 0.05) is indicated in bold; *N*: number of participants; Adj *p*: adjusted *p*-value; NS: non-significant; JD: Jordanian Dinar; PHQ-9: Patient Health Questionnaire.

Further, engagement in hobbies seemed to mitigate EE, as staff practicing hobbies reported lower EE levels (71.4%) compared to those who did not (80.3%). This association approached significance (*p* = 0.008; Adj *p* = 0.053).

In terms of marital status, single staff reported the highest levels of EE (84.3%) compared to their married (71.2%) and divorced counterparts (70%), initially suggesting a significant association (*p* < 0.001) that, however, lost strength after adjustment (Adj *p* = 0.427).

Moreover, having children appeared to offer some resilience against EE, with lower rates among those with children (67.7%) than those without (85.1%) (*p* < 0.001).

When considering monthly income, lower-income staff (<500 JD) showed elevated EE levels (75.7%), which decreased in higher-income brackets (e.g., only 59.4% for those earning >1500 JD). This pattern remains significant even after adjustment (Adj *p* = 0.004).

Experience level also played a role, with less experienced staff (<5 years) reporting higher EE levels (77%) than their more experienced counterparts (>15 years at 65.7%). Though this trend suggests that experience could buffer against EE, it did not reach statistical significance (NS) after adjustment, implying that other factors may better explain the variance in EE by experience.

The number of patients per week strongly correlated with EE, with those handling a higher patient load (>30 patients) reporting significantly higher EE (90.5%) compared to those with fewer weekly patients (66.2%) (*p* < 0.001; Adj *p* = 0.000).

Thoughts of quitting were strongly linked with EE, as staff who frequently considered leaving the field reported a considerably higher EE (93.5%) compared to those who rarely or never considered quitting (60.4% and 47.3%, respectively). This relationship remained significant even after adjustment (Adj *p* = 0.000).

Burnout workshops appear beneficial, with attendees reporting lower EE (64%) than those who had not attended (78.6%) (*p* = 0.001; Adj *p* = 0.007).

In terms of depression, depression severity correlated strongly with EE; severe depression was associated with nearly universal EE (99.1%) (*p* < 0.001). Conversely, smoking status did not significantly affect EE levels (NS), demonstrating minimal associations with this component.

#### 3.3.3. Depersonalization (DP)

Gender differences were minimal in DP, with females reporting slightly higher DP (37.6%) than males (33.3%), though this was not significant (NS), indicating that gender alone may not significantly impact DP. Age influenced DP significantly in younger staff (<30 years, 46.5%) compared to older age groups (only 17.1% for those >50 years), though this effect weakened post-adjustment (Adj *p* = 0.584). The initial significance suggests that age-related factors may influence DP.

Practicing hobbies was associated with lower DP (30.2% among hobbyists *vs*. 41.1% for non-hobbyists), a significant relationship (*p* = 0.004; Adj *p* = 0.003).

In terms of marital status, single staff exhibited higher DP (46.1%) than married (29.3%) or divorced staff (23.3%) (*p* < 0.001); an adjustment weakened this association (Adj *p* = 0.190).

Having children showed a similar buffering effect against DP, with staff without children exhibiting higher DP (45.1%) than those with children (27.2%) (*p* < 0.001).

Though monthly income did not significantly predict DP post-adjustment (Adj *p* = 0.135), middle-income groups showed higher DP (44%) than other brackets, indicating a possible interaction with financial stability.

Smoking and experience did not appear to significantly affect DP levels (NS), showing minimal associations with this burnout component. In contrast, thoughts of quitting exhibited a strong relationship with DP, as staff frequently considering quitting reported much higher DP (59.8%) than those who rarely or never did (19.8% and 12.3%) (p < 0.001). This relationship remained significant post-adjustment (Adj *p* = 0.000), underscoring a robust link between DP and job dissatisfaction.

Patient load also correlated with DP, as those managing larger weekly caseloads (>30 patients) reported markedly higher DP levels (56.7%) compared to staff seeing fewer patients (<20 at 22.1%) (*p* < 0.001; Adj *p* = 0.000).

Burnout workshops had a limited effect on DP, though participants reported slightly lower DP (25.7%) than non-participants (NS after adjustment, Adj *p* = 0.116), suggesting some benefit, albeit modest, from the workshop attendance.

Depression severity showed a strong association with DP, as staff with severe depressive symptoms reported significantly higher DP (67.9%) (*p* < 0.001), highlighting the overlap between depressive symptoms and emotional detachment from work.

#### 3.3.4. Personal accomplishment (PA)

Gender differences in PA were minimal (NS), with no clear association observed between gender and PA, indicating similar levels of accomplishment between male and female staff.

Likewise, age showed no significant effect on PA (NS), suggesting it may have limited influence on one's sense of accomplishment. Though hobbies correlated with slightly higher PA, this difference was not significant (NS), indicating that engagement in hobbies did not strongly impact PA.

In contrast, marital status showed a meaningful association with PA; single staff exhibited lower PA (33.5%) than married (22.3%) and divorced individuals (33.3%) (*p* = 0.005; Adj *p* = 0.055), suggesting that relationships might foster greater accomplishment.

Having children similarly correlated with higher PA (23.7% *vs*. 31.4% for childless staff) (*p* = 0.024), Conversely, monthly income and smoking status showed no significant association with PA (NS), suggesting that financial or lifestyle factors may not directly influence this burnout dimension. Experience also did not significantly affect PA levels (NS), further indicating that PA may not vary substantially across experience levels.

Interestingly, the number of patients per week was significantly associated with PA, as those seeing fewer patients (<20) reported higher PA (38.7%) than those with greater patient loads (e.g., 13.8% for 21–30 patients) (*p* < 0.001; Adj *p* = 0.000).

Thoughts of quitting revealed an inverse relationship with PA, where frequent contemplation of quitting correlated with lower PA (36.1%) compared to those who rarely considered it (20.9% and 22.6%) (*p* = 0.013; Adj *p* = 0.058).

Depression severity was inversely related to PA, with severe depressive symptoms being significantly associated with lower PA (44% for severe cases) (*p* < 0.001).

## 4. Discussion

Our findings highlight the pressing challenge of BO among oncology HCPs at the KHCC, using validated instruments: MBI and PHQ-9. The high levels of EE observed in this study are consistent with previous literature highlighting EE as the most common burnout domain among oncology HCPs. Emotional exhaustion is often attributed to the intense emotional engagement required to support patients with cancer, frequently culminating in “empathy fatigue” or “compassion fatigue” [22]. Potter [1] and Dugani [23] noted that empathy fatigue is prevalent among HCPs frequently exposed to patient suffering, particularly in fields like oncology where HCPs must manage the emotional complexities of end-of-life care, frequent mortality, and ongoing pain management. Continuous emotional engagement can progressively deplete HCPs' emotional resources, ultimately impairing their capacity for empathy and leading to significant EE.

The prevalence of emotional exhaustion in our study (75.7%) aligns with similar levels observed in studies conducted within Arab countries, supporting the relevance of these findings to the regional and cultural context. For instance, a systematic review by Elbarazi [11] revealed consistently high burnout rates among HCPs in Arab countries, with emotional exhaustion prevalence ranging from 50% to over 80% in certain settings. This significantly exceeds the global prevalence of nursing burnout (30.0%) (95% *CI*: 26.0%–34.0%) [24]. This discrepancy can be attributed to several factors. First, cultural differences, such as limited resources, high patient volumes, and a strong emphasis on caregiving. Second, a challenging work environment characterized by high workloads, frequent exposure to suffering, and insufficient systemic support. And third, the inherent emotional intensity of oncology care, particularly among nurses in specialized units. Further research is crucial to investigate these contextual influences and develop targeted interventions to support HCPs in high-stress environments. Furthermore, the implications of burnout extend beyond individual providers, affecting patient outcomes and healthcare systems; also, chronic emotional exhaustion can hinder HCPs' abilities to deliver optimal patient care. Emotionally exhausted providers may be more prone to making errors, struggling with empathy, or disengaging from their roles, ultimately affecting the quality of care patients receive and reducing patient satisfaction [6],[25]. This underscores the need for comprehensive support systems within oncology settings to mitigate burnout effects on patient care.

From an organizational perspective, high BO rates contribute to increased turnover, absenteeism, and recruitment challenges, all of which place additional strain on healthcare systems [26]. Reducing burnout among oncology HCPs is thus not only a matter of individual well-being but also a critical organizational priority, as high turnover and absenteeism compromise healthcare quality and continuity of care.

Our findings indicate that younger staff, particularly those under 30, experience higher levels of EE, which gradually decreases in older age groups. This trend may reflect the challenges that early-career oncology professionals face, including limited coping strategies and less experience managing emotional stressors [27]. However, this trend did not remain significant after adjustment, suggesting that other factors, such as professional experience or workload, may be more critical in moderating emotional exhaustion.

Furthermore, those with hobbies reported slightly lower levels of EE, aligning with studies that highlighted the protective effect of non-work-related activities against emotional exhaustion by providing a necessary outlet for stress relief and emotional recharge [28].

Lower income levels were also more likely to report higher levels of EE, with income remaining significant post-adjustment. Financial stress may add to the emotional burden of oncology work, suggesting the importance of adequate compensation in retaining emotionally healthy staff. Additionally, high patient load and frequent thoughts of quitting were strongly associated with elevated EE, which supports previous research indicating that workload and job dissatisfaction are primary contributors to emotional exhaustion [29]. Panagioti [7] has demonstrated the association between heavy workload and BO in healthcare settings, where task overload and inadequate rest periods compound emotional exhaustion and contribute to burnout. Interventions targeting workload reduction, adequate staffing, and access to mental health resources are essential to prevent BO. Organizational support programs, working as adaptive coping mechanisms and strategies, such as mindfulness practices, physical exercise, and peer-support systems, can enhance resilience and mitigate burnout symptoms [30],[31].

Notably, the low rate of burnout training reported by participants (19.7%) highlights a critical gap in resources available for HCPs, suggesting that regular training and support programs might help mitigate EE by providing coping strategies and emotional support. So, we suggest that training HCPs in stress-management techniques, such as cognitive-behavioral strategies and emotional regulation, may be valuable [31]. Panagioti [7] suggested that providers who adopt adaptive coping mechanisms experience lower BO rates.

The prevalence of DP, although lower than EE, remains substantial, with 35.3% of participants experiencing high levels. This form of burnout reflects a sense of emotional detachment and, in some cases, HCPs may develop a need to protect themselves from the emotional toll of constant patient interactions [22]. While temporarily effective in reducing immediate stress, long-term depersonalization risks eroding the essential therapeutic relationships that oncology care demands, where compassionate, patient-centered care is crucial [4]. This sense of detachment has broader implications, as depersonalization can foster an environment of “cold professionalism” influencing patient satisfaction and undermining the emotional support critical to the palliative needs of oncology patients.

Similar to EE, younger oncology staff exhibited higher DP levels, which could indicate that as professionals advance in their careers, they develop resilience and coping mechanisms that help manage the emotional distance without reaching detachment. Multiple studies covered similar findings on burnout, coping mechanisms, and emotional resilience in HCPs; for example, Maslach and Leiter [22] described how job-related depersonalization, especially in younger HCPs, could lead to emotional detachment. It discussed resilience as an adaptive mechanism that tends to increase with experience and length of service. Hlubocky [32] focused specifically on oncology and detailed how resilience training and experience help staff manage the emotional demands of oncology work, allowing them to maintain empathy without slipping into detachment. West [33] highlighted in his systematic review that career longevity and targeted interventions can reduce burnout symptoms, such as depersonalization, as professionals develop more effective coping mechanisms over time.

Single participants and those without children were more likely to experience DP. This aligns with research suggesting that personal relationships, especially family support, may help buffer against detachment by providing a sense of connection and purpose outside of work [34]. Salyers [34] explored the relationship between clinician burnout, including DP, and factors like family support, suggesting that strong personal connections can mitigate feelings of detachment and promote a sense of purpose outside work.

Engaging in hobbies was also associated with lower DP, reinforcing the idea that activities outside the workplace may help maintain a healthy perspective toward patient care. Additionally, workload and frequent thoughts of quitting were significant predictors of DP, underscoring the emotional impact of excessive patient load and dissatisfaction on HCPs' ability to maintain compassion in their work [35],[36].

Interestingly, training on burnout appeared to have a minor effect on DP reduction, though not significant after adjustment. A study published in Psychology Today by Maslach and Leiter [22] discussed the multifaceted nature of burnout in mental health settings, emphasizing that while training can raise awareness, it often does not significantly alleviate symptoms like depersonalization (DP). The complexity of DP may necessitate more personalized interventions, including regular counseling and peer support networks to foster emotional resilience and engagement [22],[35].

PA emerged as a complex domain, with 50.3% of participants reporting high levels but 27.2% indicating low PA. This finding suggests that while some HCPs maintain a strong sense of accomplishment, a notable portion feels unfulfilled in their work. PA is often driven by perceived impact and recognition, both of which may be limited in high-stress environments like oncology, where positive patient outcomes can be infrequent, and emotional support often goes unrecognized [36].

Reduced personal accomplishment can also lead to significant job dissatisfaction and may deter talented professionals from long-term careers in oncology, further exacerbating staffing shortages. Studies suggested that feelings of low achievement are not only demotivating but may also predict turnover, with potentially harmful impacts on oncology departments that rely on experienced and skilled providers [22]. In a field as demanding as oncology, professional fulfillment is a key component of both personal well-being and sustained commitment to care quality, underscoring the importance of organizational strategies that enhance personal accomplishment.

Participants with children and those who were married reported higher levels of PA, suggesting that family support may foster a sense of purpose and accomplishment by providing emotional resilience and stability. This aligns with a recent study published in Frontiers in Psychology that discussed the critical role of emotional resilience in enhancing well-being and employability, emphasizing the importance of personal support systems, such as family. The findings indicate that individuals with strong family support tend to experience greater emotional stability and resilience, which can lead to higher levels of personal achievement [37].

Lower patient load was also associated with higher PA, indicating that the ability to provide thorough and personalized care may enhance feelings of accomplishment. This finding aligns with previous studies that emphasized the negative impact of excessive workload on perceived job satisfaction and professional achievement [36].

The strong association between depression severity and all three burnout domains underscores the overlap between these two conditions in HCPs. Severe depressive symptoms were nearly universally associated with high EE and DP and low PA, indicating that depression may not only be a consequence of burnout but a concurrent condition that exacerbates its effects [38]. The high rates of clinically significant depressive symptoms in this study (54.5%) mirror findings in oncology staff populations worldwide, suggesting a critical need for mental health interventions targeted at reducing depressive symptoms alongside burnout [39].

The authors strongly recognize the influence of cultural norms, and region-specific interventions should be developed to encourage open discussions about burnout in a way that respects cultural values and promotes provider well-being without stigma. Faith-based support options, where appropriate, could provide an additional coping resource for HCPs who find resilience through religious practices [3],[11].

## 5. Strengths

This cross-sectional study addressed the critical issue of burnout among HCPs in oncology, a field characterized by an emotionally demanding environment. Employing a robust methodology, including the utilization of validated instruments such as the MBI and PHQ-9, and a substantial sample size of 692 HCPs, the study effectively captured the prevalence of burnout and depressive symptoms among a diverse range of HCPs, including nurses, physicians, and other allied health professionals. Furthermore, the inclusion of sociodemographic, work-related, and lifestyle factors enabled a comprehensive exploration of potential predictors of burnout and depression. Importantly, the study provided valuable insights into the unique challenges and stressors faced by oncology HCPs within the Jordanian context. The findings of this study have significant implications for improving the mental health and well-being of HCPs in oncological settings.

## 6. Limitations

As a cross-sectional study, this work cannot establish causal relationships between predictors and burnout or depression. Furthermore, relying on self-reported data introduces potential biases, such as recall bias and social desirability bias. The findings may not be fully generalizable to other healthcare settings or regions due to the specific context of the study, which was conducted at a tertiary cancer center in Jordan. Moreover, the study primarily focused on individual-level factors, potentially limiting the exploration of organizational-level factors such as leadership styles, workplace culture, and resource availability. Finally, while the study identified potential predictors, further research is needed to investigate the underlying mechanisms and develop effective interventions to address burnout and improve the mental health of oncology HCPs.

## 7. Conclusions

The findings highlight burnout as a significant issue among oncology HCPs in Jordan, especially in emotional exhaustion (EE) and depersonalization (DP), closely linked with depressive symptoms.

Future Direction: Addressing burnout requires comprehensive interventions that address both individual and job-related factors. Oncology HCPs and centers should prioritize self-care training in emotional resilience and burnout prevention, tailored to various career stages, which can equip providers with strategies for managing oncology's emotional demands. In addition, health providers should have access to mental health resources, (e.g., counseling, support groups) alongside workload management solutions, (e.g., flexible scheduling, patient load adjustments), as well as work-life balance initiatives, such as those encouraging hobbies and fostering family support. Our findings require longitudinal research to monitor burnout trends over time and assess the effectiveness of interventions in addition to incorporating qualitative components to better understand the cultural barriers to discussing mental health and burnout in Jordan. Future research may also explore the impact of pilot interventions, such as stress management workshops, on sustained reductions in emotional exhaustion, depersonalization, and depression scores among oncology HCPs. We also recommend replicating our study in diverse healthcare settings, such as general hospitals or rural clinics, to compare predictors across contexts.

## Use of AI tools declaration

The authors declare they have not used Artificial Intelligence (AI) tools in the creation of this article.
